# Escaping the trap of allergic rhinitis

**DOI:** 10.1186/s12948-015-0023-y

**Published:** 2015-08-04

**Authors:** Oliviero Rossi, Ilaria Massaro, Marco Caminati, Cristina Quecchia, Filippo Fassio, Enrico Heffler, Giorgio Walter Canonica

**Affiliations:** Allergy Unit, Azienda Ospedaliero Universitaria Careggi, Largo Brambilla 1, 50129 Firenze, Italy; Denothe Center, University of Florence, Firenze, Italy; Allergy Unit, Verona University and General Hospital, Verona, Italy; Allergy Unit, Melegnano Hospital, Melegnano (Milano), Italy and Clinical Pedagogical Laboratory and Biomedical Research, Pediatric Hospital, Brescia, Italy; Medicine Unit, ASL 3 Pistoia, Pistoia, Italy; Respiratory Medicine and Allergy - Department of Clinical and Experimental Medicine, University of Catania, Catania, Italy; Respiratory Diseases & Allergy Clinic, DIMI-Department Internal Medicine, Respiratory Diseases and Allergy Clinic, University of Genoa, IRCCS AOU S. Martino Genoa, Genoa, Italy

**Keywords:** Allergic rhinitis, Intranasal corticosteroids, Intranasal antihistamines, Therapy

## Abstract

Rhinitis is often the first symptom of allergy but is frequently ignored and classified as a nuisance condition. Ironically it has the greatest socioeconomic burden worldwide caused by its impact on work and on daily life.

However, patients appear reticent to seek professional advice, visiting their doctor only when symptoms become ‘intolerable’ and often when their usual therapy proves ineffective.

Clearly, it’s time for new and more effective allergic rhinitis treatments.

MP29-02 (Dymista®; Meda, Solna, Sweden) is a new class of medication for moderate to severe seasonal and perennial allergic rhinitis if monotherapy with either intranasal antihistamine or intranasal corticosteroids is not considered sufficient.

MP29-02 is a novel formulation of azelastine hydrochloride (AZE) and fluticasone propionate (FP). It benefits not only from the incorporation of two active agents, but also from a novel formulation; its lower viscosity, smaller droplet size, larger volume (137 μl) and wider spray angle ensure optimal coverage of, and retention on the nasal mucosa and contribute to its clinical efficacy.

In clinical trials, patients treated with MP29-02 experienced twice the symptom relief as those treated with FP and AZE, who in turn exhibited significantly greater symptom relief than placebo-patients. Indeed, the advantage of MP29-02 over FP was approximately the same as that shown for FP over placebo. The advantage of MP29-02 was particularly evident in those patients for whom nasal congestion is predominant, with MP29-02 providing three times the nasal congestion relief of FP (*p* = 0.0018) and five times the relief of AZE (*p* = 0.0001). Moreover, patients treated with MP29-02 achieved each and every response up to a week faster than those treated with FP or AZE alone and in real life 1 in 2 patients reported the perception of well-controlled disease after only 3 days. MP29-02’s superiority over FP was also apparent long-term in patients with perennial allergic rhinitis or non-allergic rhinitis, with statistical significance noted from the first day of treatment, with treatment difference maintained for a full year.

Taken together, these data suggest that MP29-02 may improve the lives of many of our patients, enabling them to finally escape the allergic rhinitis trap.

## Introduction

### The allergy trap

In Europe 150 million people are trapped by allergy [1, 2]. One out of 3 children are allergic [3] and 50 % of Europeans will suffer from allergy within the next 10 years [2]! Not surprisingly asthma and anaphylaxis get the lion’s share of medical and media attention, due to the possibility of fatality. Consequently other allergic diseases, such as allergic rhinitis (AR), are frequently ignored and often trivialised as a nuisance condition.

Ironically, AR accounts for the greatest socioeconomic burden worldwide, higher than that induced by asthma, diabetes and heart disease [4]. The knock on cost is enormous, €2 billion euros in Sweden alone [5]. Furthermore, patients with AR are daily burdened with the misery of nasal and ocular symptoms, making them tired, miserable and irritable [6]. No aspect of patients’ lives, from sleep to cognitive functions, escapes the rhinitis touch [7, 8], including an increased risk of road traffic accidents [9]. Considering that AR is also a risk factor for asthma [10], the time to ignore AR is long past.

However, patients appear reluctant to seek professional advice until their symptoms become ‘intolerable’ [11]. Unfortunately, many physicians under-estimate AR severity and under-treat it [12]. Patients, therefore, struggle to alleviate their misery, frequently use over the counter drugs and homeopathic remedies [13, 14]. Notably, many patients remain symptomatic despite optimal treatment [15] or multiple therapy usage [16, 17], the latter not endorsed by ARIA due to limited supporting evidence [18–20].

## Review

### Escaping the trap

Clearly, it’s time for new and more effective AR treatments. The last major breakthrough in symptomatic treatment was the introduction of intranasal corticosteroids (INS) over 50 years ago, now recommended as first line treatment for moderate/severe AR [21–23].

MP29-02 (Dymista®; Meda, Solna, Sweden), launched in the US in 2012 and in Europe in 2013, is a new class of medication (WHO code ATC R01AD58) for moderate to severe seasonal and perennial AR if monotherapy with either intranasal antihistamine or INS is not considered sufficient [24]. MP29-02 is a novel intranasal formulation of azelastine hydrochloride (AZE) and fluticasone propionate (FP). It benefits from the incorporation of two active agents with broad pathologic coverage and complementary effects, but also from a novel formulation. MP29-02’s lower viscosity, smaller droplet size, larger volume (137 μl) and wider spray angle ensure optimal coverage of, and retention on, the nasal mucosa contributing to its efficacy [25].

**Fig. 1:**
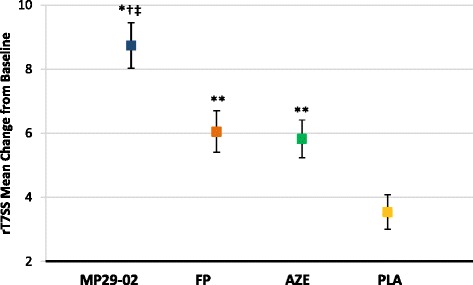
Effect of MP29-02 (*blue square*, *n* = 153), fluticasone propionate (FP; *orange square*, *n* = 151), azelastine (AZE; *green square*, *n* = 152) and placebo (PLA; *yellow square*, *n* = 151) on least squares mean change from baseline in reflective total of 7 symptom scores (rT7SS) over the entire 14-days treatment period. Precision of these estimates is indicated by the standard error. *p < 0.0001 vs PLA, ^†^ *p* = 0.0013 vs FP, ^‡^ *p* = 0.0004 vs AZE, **p ≤ 0.0017 vs PLA. Modified from Meltzer et al., (S Karger AG, Basel) [27]

The clinical development of MP29-02 has been a unique one for several reasons. Firstly it was the largest head to head development programme, to our knowledge, ever carried out in AR incorporating four 14-day seasonal AR (SAR) studies [26–28], and one long-term (52-week) study in patients with chronic rhinitis (i.e., perennial AR (PAR) and non-allergic rhinitis (NAR)) [29] (4617 patients overall). In contrast to other AR drugs, MP29-02’s efficacy was compared not only with placebo, but also versus two first-line AR treatments; a clinically-relevant study design, since in real-life patients are not treated with placebo. The MP29-02 group experienced twice the nasal and ocular symptom relief as those treated with FP or AZE alone, with all active treatments providing significantly greater symptom relief than placebo [26–28]. Indeed, the advantage of MP29-02 over commercially-available FP was approximately the same as that shown for FP over placebo (Fig. 1) [27]. MP29-02’s superiority over FP persisted long-term in patients with PAR and NAR, with statistical significance observed from the first day of treatment and sustained benefit established for a full year [29], without safety concerns [30].

In this 1-year, randomized, open-label, active-controlled, parallel-group study including more than 600 patients with allergic or non-allergic rhinitis, MP29-02 showed similar adverse events incidence (9.4 %) compared to FP (11.1 %), no evidence of late-occurring adverse events or of nasal mucosal ulcerations or septal perforations, no unusual or unexpected ocular examination findings and no clinically important laboratory findings or clinically important differences between groups in fasting AM serum cortisol levels after 12 months of treatment [30].

**Fig. 2:**
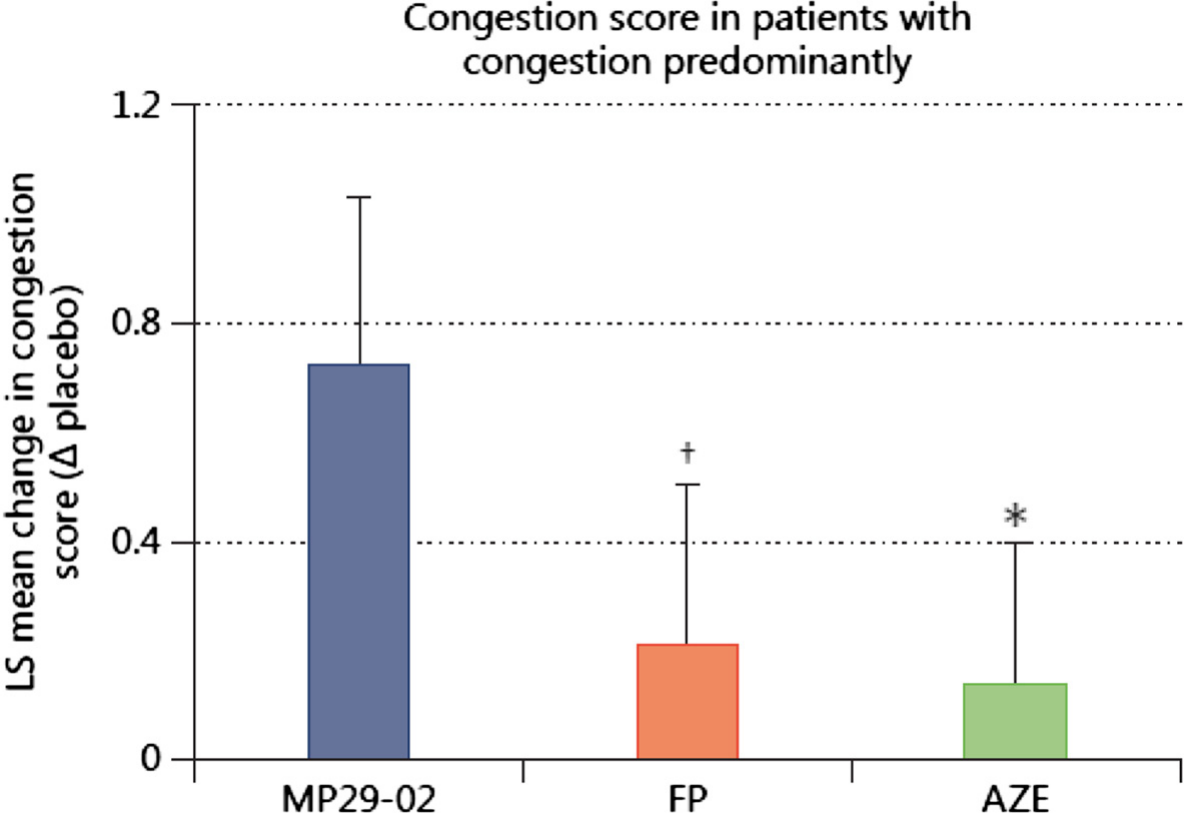
Effect of MP29-02 (*n* = 98), fluticasone propionate (FP, *n* = 84) and azelastine (AZE, *n* = 93) on nasal congestion score in those patients suffering predominantly from nasal congestion at baseline. The precision of these estimates is indicated by the upper bounds of the respective 95 % confidence intervals. * *p* ≤ 0.0001 vs MP29-02; † *p* ≤ 0.0093 vs MP29-02. Reprinted with permission from Meltzer et al., (S Karger AG, Basel) [27]

Secondly, patients recruited into the MP29-02 SAR trials [26–28] were among the most severe patients with a mean baseline reflective total nasal symptom score (rTNSS) of 19/24. They were required to have moderate to severe nasal congestion, the nasal symptom considered most bothersome [31] and often recalcitrant to treatment. These patients are usually excluded from AR clinical trials. The advantage of MP29-02 over FP and AZE was particularly evident in patients with nasal congestion predominantly, with MP29-02 providing three times the nasal congestion relief of FP (*p* = 0.0018) and five times the relief of AZE (*p* = 0.0001) (Fig. 2) [27]. By comparison, FP or AZE induced the same level of congestion relief in these patients as placebo [27]. This FP and AZE treatment failure helps to explain why many AR patients experience breakthrough symptoms and often express treatment dissatisfaction [32].

Thirdly, the efficacy of MP29-02 was assessed in a novel and clinically-relevant way by responder analyses, as suggested by the European Medicines Agency [33]. Data showed that patients treated with MP29-02 achieved each and every response (from 30 to 90 % change from baseline in rTNSS) up to week faster than those treated with FP or AZE alone [27]. It means something to patients and physicians (rather than symptom score change), enabling an estimation of the likelihood of achieving a substantial response or complete symptom relief, and when to expect that level of response. The responder analyses also identified an efficacy plateau for FP (i.e., ≥60 % response) and for AZE (i.e., ≥50 % response) above which these treatments did not significantly differ from placebo, again highlighting the unmet pharmacological need in AR and the insufficiency of currently considered first-line treatment options for many patients with moderate/severe disease. MP29-02 was significantly superior to placebo for all responses, and no plateau effect was observed [27].

Fourthly, by virtue of differently formulated active comparators, MP29-02’s development programme enabled observation of the effect of formulation on clinical efficacy. In one study [27] MP29-02 was compared to commercially-available comparators and in 3 studies [28] it was compared to FP and AZE made up in the MP29-02 formulation and delivered in the MP29-02 device. MP29-02’s treatment effect was consistent in all studies but the treatment difference was more marked in the study versus commercial comparators [27], since here the effect of formulation contributed to MP29-02’s efficacy. This may help to explain the over-additive effects of MP29-02 observed for several parameters (Fig. 2).

**Fig. 3:**
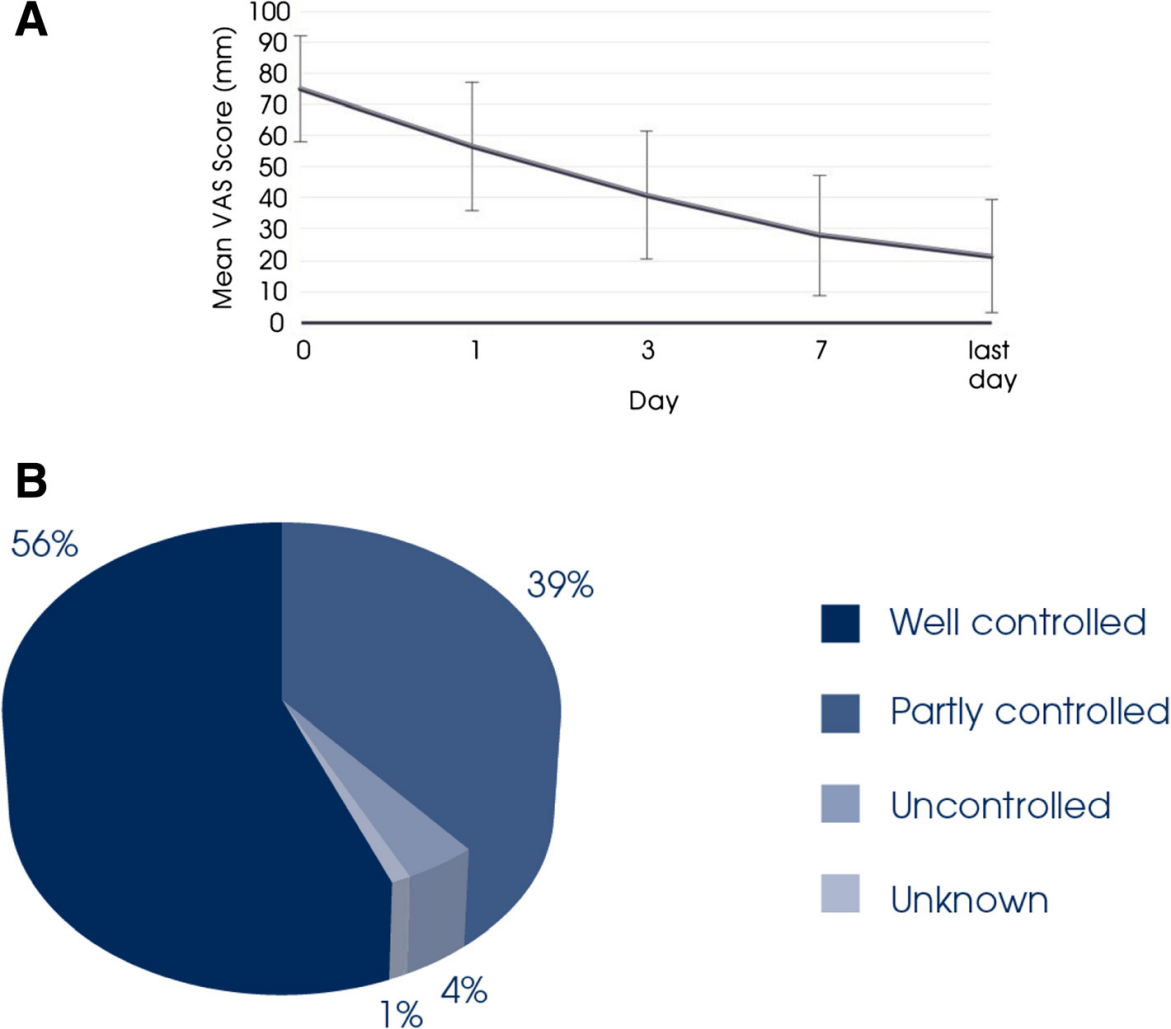
**a** Effect of MP29-02 on visual analogue scale (VAS) score over time and **b** patient perception of disease control on day 3. *N* = 1781. Reprinted with permission from Klimek et al., (Oceanside Publications, RI, USA) [34]

Finally, MP29-02’s clinical development programme has continued with a large (*n* = 1781) non-interventional real-life study [34], completing the continuum from efficacy to effectiveness. Assessment of effectiveness is crucial, since real-life studies include patients usually excluded from randomized controlled trials (RCTs; e.g., patients with co-morbid asthma, heart disease, diabetes, smokers, poorly compliant patients, etc.). Patients treated with MP29-02 in real-life experienced rapid symptom control, with 1 in 2 patients reporting the perception of well-controlled disease after only 3 days (Fig. 3) [34]. Effectiveness was assessed using a visual analogue scale (VAS) which has been recommended by MACVIA-LR ARIA as the new language of AR control and will form the basis of the new AR guideline termed the AR integrated care pathway [35]. The real-life response to MP29-02, was better than that observed in the RCTs, independent of disease severity or phenotype (i.e., PAR, SAR or PAR + SAR) [34].

## Conclusions

MP29-02 provides significantly better nasal and ocular symptom relief than INS, which up to now has been considered the most effective treatment for AR. Clinical data analyses showed that, unlike other therapies, MP29-02 has no efficacy threshold, and continues to provide superior relief to established therapies regardless of patient type, season, symptom or severity. Its effectiveness has been proven in real life, with response rates even greater than those observed in controlled trials. Taken together, these data suggest that MP29-02 may improve the lives of many of our AR patients, enabling them to finally escape the AR trap.

## Abbreviations

AR: Allergic rhinitis
ARIA: Allergic Rhinitis and its Impact on Asthma
AZE: Azelastine hydrochloride
FP: Fluticasone propionate
INS: Intranasal corticosteroids
MACVIA: contres les MAladies Chronique pour un VIeillissement Actif
NAR: Non allergic rhinitis
PAR: Perennial allergic rhinitis
RCT: Randomized controlled trials
SAR: Seasonal allergic rhinitis
VAS: Visual analogue scale
